# Evidence of stable genetic structure across a remote island archipelago through self-recruitment in a widely dispersed coral reef fish

**DOI:** 10.1002/ece3.260

**Published:** 2012-12

**Authors:** Mark A Priest, Andrew R Halford, Jennifer L McIlwain

**Affiliations:** 1 University of Guam Marine LaboratoryMangilao, Guam 96923; 2 Red Sea Research Center, King Abdullah University of Science and TechnologyThuwal, 23955, Saudi Arabia; 3 Department of Environment and Agriculture, Curtin UniversityGPO Box U1987, Perth, WA, Australia

**Keywords:** Connectivity, Guam, Micronesia, population genetics, self-recruitment, *Siganus spinus*

## Abstract

We used microsatellite markers to assess the population genetic structure of the scribbled rabbitfish *Siganus spinus* in the western Pacific. This species is a culturally important food fish in the Mariana Archipelago and subject to high fishing pressure. Our primary hypothesis was to test whether the individuals resident in the southern Mariana Island chain were genetically distinct and hence should be managed as discrete stocks. In addition to spatial sampling of adults, newly-settled individuals were sampled on Guam over four recruitment events to assess the temporal stability of the observed spatial patterns, and evidence of self-recruitment. We found significant genetic structure in *S. spinus* across the western Pacific, with Bayesian analyses revealing three genetically distinct clusters: the southern Mariana Islands, east Micronesia, and the west Pacific; with the southern Mariana Islands being more strongly differentiated from the rest of the region. Analyses of temporal samples from Guam indicated the southern Mariana cluster was stable over time, with no genetic differentiation between adults versus recruits, or between samples collected across four separate recruitment events spanning 11 months. Subsequent assignment tests indicated seven recruits had self-recruited from within the Southern Mariana Islands population. Our results confirm the relative isolation of the southern Mariana Islands population and highlight how local processes can act to isolate populations that, by virtue of their broad-scale distribution, have been subject to traditionally high gene flows. Our results add to a growing consensus that self-recruitment is a highly significant influence on the population dynamics of tropical reef fish.

## Introduction

For most marine organisms, a pelagic larval stage provides the primary mechanism for dispersal among spatially fragmented habitat patches (Kritzer and Sale 2004; Cowen and Sponaugle 2009). The degree to which larvae disperse and populations are connected has a profound influence on the population dynamics of a species (Hixon et al. 2002; Gaines et al. 2007), with the stability and resilience of populations dependent upon a constant supply of larvae, either locally or externally sourced (Warner and Cowen 2002). Despite the obvious need, quantification of connectivity has proven difficult. This is principally due to the small size of larvae, their patchy distribution, and high rates of larval mortality (Leis 1991), all of which has severely hindered attempts at in situ studies.

Because the pelagic larval duration of many fish larvae can be more than one month (Victor and Wellington 2000), coral reef fish populations were assumed to be demographically open with a large dispersive potential (Roughgarden et al. 1985; Caley et al. 1996). In addition, studies on settlement-stage larval fish behavior have shown that many species are strong swimmers (Stobutzki and Bellwood 1997; Fisher 2005) and can respond to a variety of potential settlement cues, both olfactory (Atema et al. 2002; Lecchini et al. 2005) and auditory (Leis et al. 2002; Simpson et al. 2005). The combination of these traits was thought to favor maximization of an individual's dispersal distance. However, over the past 10 years studies have shown that these same traits can equally be used to actively reduce dispersal and entrain larvae close to natal reefs (Gerlach et al. 2007).

Traditionally, the most widely used approach for estimating connectivity has been population genetics (see reviews by Hellberg 2007; Jones et al. 2009). The analysis of genetic variation among spatially isolated populations allows for an indirect assessment of connectivity (Neigel 1997). However, in many marine organisms gene flow is high over evolutionary timescales, with only a few successful migrants per generation needed to produce genetic homogeneity among populations (Slatkin 1993). This results in connectivity estimates that reflect historical processes over multiple generations (Hellberg 2007). Additionally, population-level estimates of connectivity require several simplifying assumptions, which may be hard to satisfy in natural biological systems (Whitlock and McCauley 1999). Such limitations have recently been overcome with the development of new, more powerful analyses using highly variable microsatellite markers that have shifted the focus of analysis from populations to individuals (Balloux and Lugon-Moulin 2002; Pearse and Crandall 2004). In addition to using more variable markers, advances in statistical methodologies using maximum likelihood and Bayesian inference enables sorting of genetically similar individuals into discrete populations, and also enables individuals to be assigned to their population of origin (Manel et al. 2005), thus enumerating rates of contemporary connectivity (e.g., Underwood et al. 2007; Saenz-Agudelo et al. 2009). Direct estimates can also be obtained using parentage analysis (Planes et al. 2009), which has been shown to provide estimates of self-recruitment identical to tagging studies (Jones et al. 2005). While parentage analysis was once restricted to fish species in which all prospective parents could be sampled (see e.g., Jones et al. 2005; Planes et al. 2009), novel Bayesian parentage methods now make it possible to assess parentage when only a small proportion of potential parents are sampled (Christie 2010).

Utilizing microsatellite markers and advances in statistical capabilities, research efforts in recent years have seen our understanding of population connectivity in reef fish increase dramatically. Studies have documented high levels of self-recruitment in species with large potential dispersive capacity (Jones et al. 1999, 2005; Almany et al. 2007). No populations, however, have been found to be completely closed (Jones et al. 2009). Larvae have been shown to disperse up to 35 km away from natal sites in populations with high local retention rates (Planes et al. 2009), and extensive gene flow between populations is commonly observed (van Herwerden et al. 2003; Purcell et al. 2006; Horne et al. 2008; Christie et al. 2010). Consequently, coral reef fish populations cannot be categorized as merely open or closed (Cowen et al. 2000; Mora and Sale 2002) with many populations appearing to exhibit both self-recruitment and long-range dispersal, the ratio of which may vary dynamically with location and time (Cowen and Sponaugle 2009).

To date, the focus of most connectivity studies has been quantification of spatial patterns with little knowledge of the temporal stability of demographically relevant connectivity (but see Selkoe et al. 2006; Jones et al. 2010). However, discrete cohorts can possess unique genetic signatures (Planes and Lenfant 2002) and examination of the variability of these genetic signatures over time can lead to insights into the nature of larval dispersal. One process that may dramatically affect temporal gene flow is sweepstakes reproductive success, where only a small proportion of reproductive adults are responsible for the majority of the recruitment within a population (Li and Hedgecock 1998; Hedgecock et al. 2007). This reduced genetic contribution to subsequent recruitment leads to testable hypotheses regarding genetic diversity and relatedness within and among recruits and adult populations (Hedgecock 2010). Analysis of multiple dispersal events are needed to resolve and understand the complex larval dispersal patterns of coral reef fish, which can ultimately be used to increase our understanding of population dynamics and lead to better resource management (Selkoe et al. 2006; Planes et al. 2009; Christie et al. 2010).

**Figure 1:**
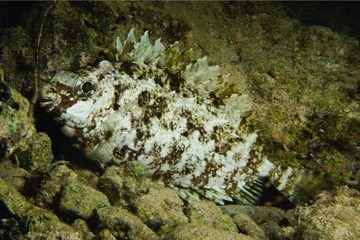
An adult scribbled rabbitfish *(Siganus spinus)* pictured at night in Piti Marine Preserve, Guam. Photo by Mark Priest.

In this study, we investigate the population structure of the scribbled rabbitfish (*Siganus spinus*) in the western Pacific, with a particular focus on the islands of the southern Mariana archipelago (Fig. 1). This herbivorous species is a highly fecund, fast-growing, common inhabitant of shallow coral reefs throughout the Indo–Pacific region (Woodland 1990). On Guam, the largest and most southerly island in the Mariana chain, settlement-stage *S. spinus* recruit to shallow reef flats in large numbers, usually twice a year (Tsuda and Bryan 1973), at the end of a 32-day pelagic phase (Chirichetti 1996). These recruits settle at a relatively large size, approximately 43-mm fork length (FL) (Kami and Ikehara 1976), and have strong swimming capabilities (Fisher 2005). Newly settled recruits are considered a cultural delicacy and are harvested en masse by local residents. Adult *S. spinus* are also a favored food fish heavily targeted by spear and net fishermen. Recent declines in Guam's reef fish stocks (Newton et al. 2007; Zeller et al. 2007) and uncertainties over population size and stock structure have seen Guam's rabbitfish populations recognized as a “species group of greatest conservation need” by the local fisheries agency (Bassler and Aguon 2006). Should self-recruitment be a significant pathway for population replenishment, any decrease in spawner biomass as a result of overharvesting could have considerable effects on subsequent recruitment events and the long-term sustainability of the fishery (Man et al. 1995).

Here, we use six microsatellite markers to investigate (1) the spatial genetic population structure of *S. spinus* at regional (western Pacific) to local (southern Mariana islands) scales. Prevailing large-scale current patterns indicate an east–west flow between islands in this region and analysis of gene flow pathways will identify any genetic discontinuities and possible barriers to dispersal. The lack of any obvious physical barriers would implicate oceanographic conditions in such a case. Sampling from multiple locations around Guam provides insights into whether significant structure is evident at the island scale. Such information is important in the context of formulating effective conservation management plans for this species; (2) in conjunction with the local-scale spatial sampling we also sampled genetic variability within and between multiple cohorts at several sites on Guam, to assess the temporal stability of gene flow, degree of self-recruitment, and evidence of sweepstakes reproductive success. Many connectivity studies on coral reef fish that have included temporal sampling have focused on small, long-lived site-attached fish (see e.g.*,*Hepburn et al. 2009; Planes et al. 2009; Christie et al. 2010; Jones et al. 2010), whereas this study provides one of the first assessments of a more mobile reef fish species.

## Methods

### Sampling regime

**Figure 2:**
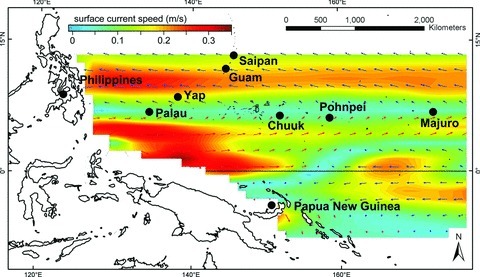
Sampling locations for *Siganus spinus* across the western Pacific. For detailed locations of sites and subsites, see Table 1. Prevailing regional surface current direction is denoted by arrows with speed represented in color. Current data are long-term mean (1993–2009) obtained from the NOAA ocean surface current analyses—real time website (http://www.oscar.noaa.gov).

**Figure 3:**
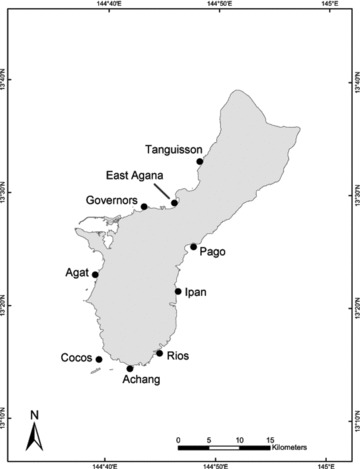
Sampling sites for adult and recruit collections of *Siganus spinus* from Guam. See Table 2 for sample size and time of collections. Sampling was not conducted on the northeast of Guam as there is no suitable reef flat habitat.

To assess spatial genetic structure at regional scales (200–5000 km), tissue samples from 971 *S. spinus* individuals were collected from nine islands across the western Pacific (Fig. 2; Table 1) between August 2007 and November 2009. Additionally, where possible, individuals were collected from at least two discrete sites at each island to test for population structure at local (within island, 1–60 km) spatial scales. Individuals were sampled across the whole size range of the species (>60 mm FL) to capture as much genetic diversity of the population as possible, and to minimize the influence of any single, potentially genetically atypical, large cohort. To assess temporal genetic structure on Guam, 331 newly settled *S. spinus* recruits (<50 mm FL) were collected from four discrete recruitment events: November 2007, July 2008, September 2008, and October 2008 from nine locations (Fig. 3; Table 2). The predictable timing of recruitment (±3 days over the last quarter moon; Kami and Ikehara 1976) allowed separate cohorts of recruits to be clearly identified as belonging to discrete recruitment events. However, the variable nature of recruitment meant individuals could not be sampled from every site for each recruitment event.

**Table 1:** Summary of *Siganus spinus* sampling locations, site codes, sample sizes (*N*), and genetic diversity measures over all loci for the large-scale spatial analyses. Observations include mean number of alleles (*N*_A_), number of private alleles (*N*_PA_), allelic richness (*AR*), observed (*H*_O_) and expected (*H*_E_) heterozygosity, and mean inbreeding coefficient (*F*_IS_)

			Location								
										
Island	Site	Site code	Latitude	Longitude	*N*	*N*_A_	*N*_PA_	*AR*	*H*_O_	*H*_E_	*F*_IS_
Philippines	Dumaguete	PI_D_	9.3194	123.3137	88	7.60	1	4.53	0.42	0.44	0.17
Palau	Babeldaob	PA_B_	7.3191	134.5331	50	7.80	1	4.56	0.39	0.44	0.22
	Korror	PA_K_	7.2999	134.4885	40	6.60	1	3.83	0.41	0.44	0.12
Yap	Wreck	YA_W_	9.4966	138.1514	26	5.60	0	3.63	0.41	0.47	0.24
PNG	Kimbe Bay	PN_K_	–5.363	150.2381	9	4.60	0	3.27	0.40	0.48	0.14
Guam	Cocos	GU_C_	13.2592	144.6572	26	5.40	0	3.01	0.35	0.39	0.12
	Governors	GU_G_	13.4801	144.7269	39	5.00	0	3.32	0.28	0.37	0.20
	Ipan	GU_I_	13.3585	144.773	67	6.80	0	3.37	0.34	0.39	0.21
	Pago	GU_P_	13.4275	144.7971	28	5.60	0	2.99	0.35	0.39	0.13
	Tanguisson	GU_T_	13.5434	144.8075	87	6.40	0	3.42	0.31	0.38	0.07
Saipan	Coral Ocean	SA_C_	15.1082	145.7063	18	5.00	0	2.61	0.37	0.39	0.06
	Laulau	SA_L_	15.1604	145.7588	56	5.80	0	3.32	0.34	0.39	0.22
	Wing Beach	SA_W_	15.2734	145.7907	19	5.00	0	2.87	0.34	0.42	0.20
Chuuk	Peniya	CH_P_	7.4567	151.8882	92	6.60	0	3.89	0.36	0.43	0.17
	Xavier	CH_X_	7.4439	151.8864	51	5.80	0	3.36	0.35	0.44	0.14
Pohnpei	Sokeh	PO_S_	6.969	158.1556	114	6.40	0	3.68	0.34	0.42	0.09
	Nan Modal	PO_N_	6.8147	158.3186	108	6.40	0	3.70	0.37	0.44	0.12
Majuro	Airport	MA_A_	7.0605	171.2611	53	5.40	0	3.43	0.34	0.42	0.21

PNG, Papua New Guinea.

**Table 2:** Summary of *Siganus spinus* sampling locations, times, and sample sizes for Guam

		Recruitment event				
			
Site	Site code	November 2007	July 2008	September 2008	October 2008	Adult samples >100 mm FL^1^
Achang	Ach	20	–	–	–	–
East Agana	EAg	16	–	21	–	–
Cocos	Coc	–	–	–	–	24
Agat	Aga	13	–	20	–	–
Governors	Gov	–	–	26	20	26
Ipan	Ipa	13	–	19	–	42
Pago	Pag	–	–	22	–	11
Rios	Rio	–	15	48	8	–
Tanguisson	Tan	9	19	19	23	33

1 Includes individuals >100 mm FL used in large-scale spatial analyses.

All fish were collected from shallow reef-flats using hand spears, with the exception of the Philippine samples, which were obtained from the Dumaguete fish market. Individuals were kept in ice slurry before being processed. Tissue samples, taken as fin clips, were preserved in 95% ethanol (EtOH) and stored at room temperature while awaiting further analysis.

### DNA extraction and laboratory analysis

Genomic DNA was extracted using DNeasy Blood and Tissue kits (QIAGEN, Valencia, CA, USA). Samples were genotyped at five microsatellite loci originally designed for the *S. spinus* congener *S. fuscescens* (Ravago-Gotanco et al. 2010). The loci used in this study were *Sfus-8, Sfus-9, Sfus-56, Sfus-98,* and *Sfus-113*. Samples from Guam were also genotyped for the additional marker *Sfus-21*. Polymerase chain reactions (PCR) were carried out individually in a 10 µl reaction volume containing: 1 µl 10× Taq polymerase buffer, 0.2 µl Taq DNA polymerase, 0.2 µl 10mM dNTPs, 0.5 µl 25mM MgCl_2_, 5.1 µl ddH_2_O, 1 µl of genomic DNA (∼10–40 ng µl^–1^), and 1 µl each of forward and reverse primers. For loci *Sfus-8, Sfus-21, Sfus-56,* and *Sfus-113*, primer concentrations were 5 µM; for locus *Sfus-98*, primer concentration was 2.5 µM. Thermocycling conditions for the PCRs consisted of an initial step of 80°C for 1 min and a denaturing step of 94°C for 2 min. Ten cycles of 30 sec at 94°C, 30 sec at 56°C, and 30 sec at 72°C were followed by 20 cycles of 30 sec at 94°C, 30 sec at 54°C, and 30 sec at 72°C, with a final extension step of 4 min at 72°C. Capillary electrophoresis was performed on pooled PCR products using an AB 3730 DNA Analyzer (Applied Biosystems, Carlsbad, CA, USA), and scored with GeneMapper v. 3.7 (Applied Biosystems, Carlsbad, CA, USA). All PCR reactions and genotyping were performed by the Australian Genome Research Facility, Brisbane, Australia.

### Genetic analyses

The mean number of alleles per locus, allelic richness, and number of observed alleles were calculated using fstat v. 2.9.3 (Goudet 2001). Expected and observed heterozygosities, and the number of private alleles per population, were evaluated using GenAlEx v. 6.1 (Peakall and Smouse 2006). Departures from Hardy–Weinberg equilibrium (HWE) within all populations by locus, and across all loci were examined using genpop v. 3.4 (Raymond and Rousset 1995); significance levels were adjusted with sequential Bonferroni correction for multiple tests for *P* < 0.05. To assess the cause of any deviations from HWE, micro-checker (van Oosterhout et al. 2004) was used to assess the data for null alleles, stuttering, and large-allele dropout. Loci were tested for linkage disequilibrium using genpop (1000 batches, 1000 iterations) for each locus pair across all populations. One hundred and twenty-eight samples were regenotyped to determine the study-specific error rate and resolve suspected null homozygotes.

### Large-scale population structure and assignment tests

Population structure was assessed using standard *F*_ST_ genetic differentiation measures and Bayesian assignment methods. Global and population pairwise *F*_ST_ values between all sampled sites were calculated using FreeNA (Chapuis and Estoup 2007), with and without correction for null alleles, and assessed using Fisher's exact tests of significance including sequential Bonferroni correction for multiple tests for *P* < 0.05 with fstat. Regional genetic structure was assessed at the island level using analysis of molecular variance (AMOVA) (Excoffier et al. 1992) implemented in arlequin v. 3.11 (Excoffier et al. 2005). Significance of *F*_ST_ values was calculated using 10,000 Markov Chain Monte Carlo (MCMC) permutations of alleles across clusters, also using arlequin. Results were visualized by performing a principle coordinate analysis (PCoA) on a genetic distance matrix constructed using pairwise *F*_ST_ values. Patterns of isolation-by-distance were investigated by plotting genetic distance against geographic distance and tested with a mantel test using 999 permutations implemented in GenAlEx. Cavalli-Sforza and Edwards’ (1967) chord distance (*D*_C_) was used as it is insensitive to departures from HWE. Geographic distances were calculated as the shortest distance by sea between the midpoints of sampling locations.

To investigate the presence of subtle genetic structure and spatial genetic discontinuities, we used Bayesian assignment methods to determine the most likely number of homogenous genetic clusters of all sampled individuals. First, structure v. 2.2.3 (Pritchard et al. 2000; Falush et al. 2007) was used to assign individuals to a predetermined number of subgroups (*K*) based on the likelihood of the individual belonging to each subgroup, as determined by the individual's allele frequencies at each locus. *K* was set from 1 to 18 and each model run was repeated independently 10 times using a run length of 100,000 MCMC repetitions and a burn-in period of 100,000. The model was run with admixture, correlated allele frequencies, and with prior location options (Hubisz et al. 2009). The most appropriate *K* was determined from plots of ad hoc posterior probability models of Δ*K*, as extracted by structure harvester v. 0.56.4 (Evanno et al. 2005; Earl and von Holdt 2011). Second, the geneland v. 3.2.1 (Guillot et al. 2005, 2008) package for the *R* statistical software environment (R Development Core Team 2009) was employed to assess spatial genetic discontinuities between sampling locations. geneland uses a Bayesian clustering model similar to structure, but also includes geographic sampling location data as a weak prior to produce spatially explicit maps of genetically homogenous clusters. Again, 10 independent runs were used for each *K*, with *K* ranging from 1 to 18, using 1,000,000 MCMC repetitions with a burn-in period of 200,000 and a thinning value of 1000.

In order to assess the origin of Guam's recruits, we used the Bayesian assignment method of Rannala and Mountain (1997) implemented in geneclass2 (Piry et al. 2004) using the clusters detected by structure as source populations. Recruits were assigned to a population if the probability of assignment was greater than 0.05 to only one source population, as determined using the simulation algorithm of Paetkau et al. (2004) (10,000 simulations).

### Fine-scale spatial structure and temporal comparisons

To assess fine-scale spatial and temporal patterns of genetic differentiation on Guam, AMOVA was used in arlequin to partition the genetic variance between location and life-history stage, that is, adults and recruits. This evaluates genetic differences based on allele frequency variation between all samples. We also constructed a pairwise matrix of relatedness values between all adult and recruit samples from Guam using Queller and Goodnight's relatedness metric (Queller and Goodnight 1989), *R*_XY_, implemented in GenAlEx. This metric describes the relationship of pairs of individuals based on the number of shared alleles standardized by allelic state. Adults were classified as individuals >100 mm FL, as this is the size at first maturity (unpubl. data). PCoA was used to visualize the results and nonparametric multivariate analysis of variance (MANOVA) (Anderson 2001; McArdle and Anderson 2001) was used to test whether individuals within sample groups shared more alleles with each other than individuals of other groups. Significance was assessed using 999 permutations, implemented in primer v. 6 (Clarke and Gorley 2006). Tests were performed on all adult and recruit samples for differences between sample site and life history, and a subset of data, containing only the four sites at which both adult and recruit samples were obtained, was reanalyzed. All recruit samples were also tested for differences between recruitment events using nonparametric MANOVA. Differences in heterozygosity between adult and recruit samples within all sites and recruitment events were assessed in fstat using 10,000 permutations. One would expect increased relatedness (i.e., greater shared alleles) within cohorts and decreased relatedness between cohorts if sweepstakes reproduction is evident, as a result of the high variance of an individuals’ chance of successfully reproducing. Similarly, one would also expect to see decreased heterozygosity in recruit populations compared to adult populations (Hedgecock 2010).

Finally, all recruit samples were added to the region-wide dataset and PCoA was performed on pairwise *F_ST_* values. Samples were grouped into the three clusters determined by the region-wide analyses, including Guam recruits as a fourth cluster, and pairwise comparisons were made between all clusters using nonparametric MANOVA in primer.

## Results

Genetic diversity estimates were generally low for all sample groups. Mean number of alleles per locus within sample groups varied from 4.6 to 7.8. The total number of alleles at each locus was highly variable, ranging from 23 (*Sfus-8*) to 2 (*Sfus-56* and *Sfus-9*). Both *Sfus-56* and *Sfus-9* were monomorphic within some sample groups. Allelic richness ranged from 2.61 to 4.56 and was generally lower in the Guam recruit samples than the region-wide samples. A total of three private alleles were present in the dataset (Table 1). Exact tests for linkage disequilibrium across all sample groups found no loci pair was in linkage disequilibrium and that loci could be treated as independent. Replicate genotyping of 128 sampled showed high levels of repeatability (>96% agreement).

Significant departures from HWE were detected at locus *Sfus-8* in 36 of the 40 sample groups (Tables A1 and A2). Of the other five loci, none exhibited more than three departures from HWE across all 40 sample groups. Results from the program micro-checker suggest that deviations from HWE in *Sfus-8* are likely due to the presence of null alleles, which resulted in heterozygote deficiencies. The effect of null alleles on population differentiation estimates was assessed by analyzing the data with FreeNA (Chapuis and Estoup 2007), with and without correction for null alleles. Global *F*_ST_ values showed minimal differences between these two datasets (noncorrected *F*_ST_= 0.014; null allele corrected *F*_ST_= 0.015) and analysis of pairwise *F*_ST_ estimates showed no differences between either dataset. Bayesian analyses using datasets including and omitting *Sfus-8* also resulted in congruent outcomes, indicating the presence of null alleles had no tangible effect on the results.

**Table 3:** Pairwise *F_ST_* estimates between 18 sampled locations for *Siganus spinus* for five microsatellite loci (lower diagonal) and corresponding *P* values (upper diagonal). Estimates in bold typeface are significant (*P* < 0.05 after Bonferroni correction)

Sample	CH_P_	CH_X_	GU_C_	GU_G_	GU_I_	GU_P_	GU_T_	MA_A_	PA_B_	PA_K_	PI_D_	PN_K_	PO_S_	PO_N_	YA_W_	SA_C_	SA_L_	SA_W_
CH_P_	–	0.471	**0**.**000**	**0**.**000**	**0**.**000**	**0**.**000**	**0**.**000**	0.007	0.009	**0**.**000**	**0**.**000**	0.140	0.322	0.005	0.071	**0**.**000**	**0**.**000**	**0**.**000**
CH_X_	0.002	–	**0**.**000**	**0**.**000**	**0**.**000**	0.001	**0**.**000**	0.012	0.034	0.002	**0**.**000**	0.124	0.555	0.091	0.283	0.001	**0**.**000**	**0**.**000**
GU_C_	0.037	0.059	–	0.171	0.802	0.819	0.384	**0**.**000**	**0**.**000**	**0**.**000**	**0**.**000**	0.131	**0**.**000**	**0**.**000**	0.002	0.656	0.713	0.340
GU_G_	0.034	0.056	0.003	–	0.400	0.432	0.880	0.004	**0**.**000**	**0**.**000**	**0**.**000**	0.027	0.002	**0**.**000**	0.006	0.209	0.552	0.104
GU_I_	0.038	0.059	0.000	0.004	–	0.159	0.559	**0**.**000**	**0**.**000**	**0**.**000**	**0**.**000**	0.031	**0**.**000**	**0**.**000**	**0**.**000**	0.392	0.816	0.418
GU_P_	0.036	0.055	0.000	0.000	0.003	–	0.705	0.003	0.004	**0**.**000**	**0**.**000**	0.134	0.001	0.001	0.021	0.718	0.591	0.594
GU_T_	0.047	0.065	0.009	0.007	0.013	0.004	–	**0**.**000**	**0**.**000**	**0**.**000**	**0**.**000**	0.007	**0**.**000**	**0**.**000**	**0**.**000**	0.191	0.945	0.294
MA_A_	0.024	0.039	0.016	0.011	0.016	0.012	0.011	–	0.008	**0**.**000**	**0**.**000**	0.153	0.532	0.543	0.030	0.001	**0**.**000**	0.001
PA_B_	0.010	0.013	0.035	0.041	0.031	0.035	0.043	0.020	–	0.604	0.193	0.942	0.002	0.004	0.863	0.021	**0**.**000**	0.002
PA_K_	0.012	0.019	0.051	0.059	0.057	0.051	0.058	0.034	0.005	–	0.209	0.033	**0**.**000**	0.001	0.237	**0**.**000**	**0**.**000**	**0**.**000**
PI_D_	0.019	0.026	0.043	0.047	0.041	0.042	0.051	0.029	0.003	0.004	–	0.044	**0**.**000**	**0**.**000**	0.010	**0**.**000**	**0**.**000**	**0**.**000**
PN_K_	0.033	0.048	0.025	0.045	0.019	0.027	0.042	0.014	0.003	0.041	0.021	–	0.222	0.236	0.794	0.134	0.007	0.198
PO_S_	0.012	0.022	0.016	0.013	0.014	0.010	0.015	0.000	0.011	0.022	0.019	0.011	–	0.638	0.090	0.001	**0**.**000**	**0**.**000**
PO_N_	0.003	0.005	0.032	0.029	0.031	0.028	0.040	0.014	0.005	0.016	0.018	0.015	0.006	–	0.081	**0**.**000**	**0**.**000**	**0**.**000**
YA_W_	0.007	0.014	0.021	0.026	0.026	0.018	0.029	0.004	0.002	0.004	0.005	0.000	0.001	0.002	–	0.028	**0**.**000**	0.003
SA_C_	0.045	0.060	0.001	0.015	0.007	0.008	0.011	0.017	0.030	0.053	0.035	0.019	0.020	0.036	0.017	–	0.255	0.709
SA_L_	0.044	0.061	0.000	0.001	0.004	0.000	0.000	0.010	0.041	0.061	0.052	0.032	0.014	0.037	0.024	0.004	–	0.395
SA_W_	0.050	0.061	0.012	0.011	0.004	0.013	0.016	0.017	0.037	0.072	0.052	0.022	0.021	0.037	0.027	0.004	0.003	–

### Large-scale spatial population structure

**Figure 4:**
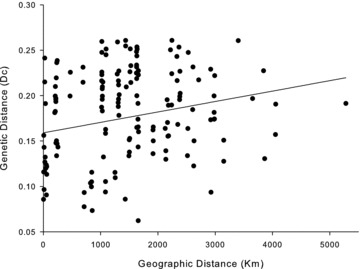
Relationship between geographic (Km) and genetic distance (*D*_C_) of *Siganus spinus* among 18 sampled sites across the western Pacific. Points represent comparisons between pairs of sample groups.

**Figure 5:**
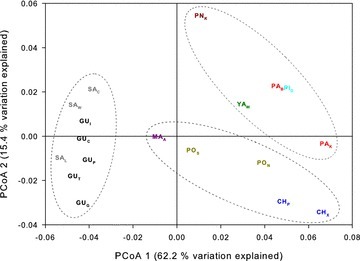
Principal coordinate analysis of all pairwise *F*_ST_ comparisons between the 18 sites sampled in the western Pacific. Both axes combined explain 77.6% of the total variation. See Table 1 for site codes. Samples are color coded by island. Dashed rings denote population clusters detected by Bayesian analyses.

There was considerable genetic differentiation across the study region (*F*_ST_= 0.023, *P* < 0.001) with 41% of pairwise *F*_ST_ comparisons significant (Table 3). A significant isolation-by-distance relationship was detected throughout the study region (Fig. 4) (linear regression: *R*^2^= 0.068; *P*= 0.013, Mantel test), with genetic distance increasing with greater geographical separation. Moreover, at the within-island scale none of the 16 pairwise *F*_ST_ estimates was significantly different from zero, indicating little genetic differentiation within islands. When samples were grouped at the island level, AMOVA showed a significant proportion of genetic variance (1.7%, *P* < 0.001) was partitioned among islands (Table 4A). This was also supported by a PCoA of pairwise *F*_ST_ values, where most within-island samples clustered closer to each other than between-island samples (Fig. 5). The PCoA analysis also revealed a significant difference between samples from the southern Mariana Islands (Guam and Saipan) and the rest of the study area (nonparametric MANOVA: *F*= 57.71, *P*= 0.001), with the southern Mariana samples grouping out along PCoA Axis 1.

**Table 4:** Summary of the analysis of molecular variance used to examine spatial and temporal genetic structure of *Siganus spinus*

	Source of variation	S.S.	df	%	*P*
Large-scale spatial comparison					
(A) Grouped by island	Among Islands	41.91	8	1.74	<**0**.**001**
	Among sites within islands	12.22	9	0.02	0.425
	Among individuals within sites	1272.48	953	22.20	<**0**.**001**
	Within individuals	818.50	971	76.04	**0**.**001**
Fine-scale spatial and temporal comparisons					
(B) Guam samples grouped by	Among adults and recruits	1.35	1	0.00	0.554
life-history stage	Among sites within adults and recruits	30.31	20	0.00	0.498
	Among individuals within sites	674.50	445	17.11	<**0**.**001**
	Within individuals	501.00	467	82.89	<**0**.**001**
(C) Recruits grouped recruitment	Among recruitment events	4.14	3	0.00	0.784
event	Among sites within recruitment events	20.76	13	0.18	0.368
	Among individuals within sites	473.89	314	18.06	<**0**.**001**
	Within individuals	346.50	331	81.76	<**0**.**001**

S.S., sum of squares; df, degrees of freedom; %, amount of variation explained by specific component; *P* indicates significance (values <0.05 are shown in bold).

**Figure 6:**
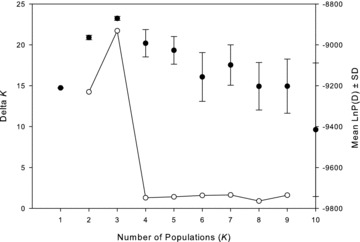
Large-scale spatial population structure detected in *Siganus spinus* using the structure program. The peak in Δ*K* (open circles) corresponds to the number of homogenous populations (*K*) detected from 10 independent runs of each model. Δ*K* is an ad hoc statistic based on the rate of change of log-likelihood as *K* is increased. Mean LnP(D) (closed circles) is the mean of 10 independent runs.

**Figure 7:**
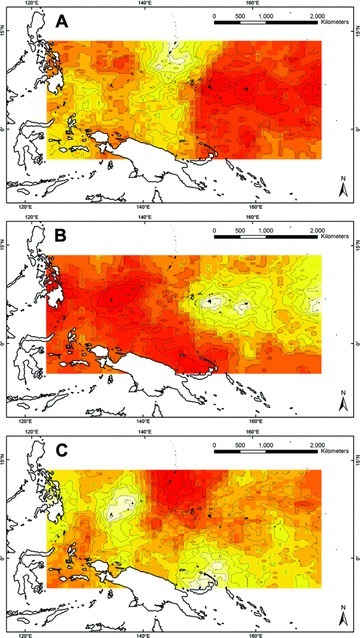
Population structure of *Siganus spinus* in the western Pacific detected using the spatially explicit clustering model in geneland. Each panel (A–C) represents the posterior probabilities of membership of one of the four populations detected in the analysis. White to red indicates high to low probability of membership of the specific cluster.

Bayesian analyses used to detect genetic clustering returned results congruent with PCoA. structure revealed three genetically distinct clusters in the data (Fig. 6). Further Bayesian-based analyses with geneland produced similar results to structure. Plots of the posterior distribution of the estimated number of populations indicated three populations were present in the data, with geneland returning the same clustering pattern as structure (Fig. 7A–C): Guam and Saipan (southern Mariana Islands); Chuuk, Pohnpei, and Majuro (East Micronesia); and the Philippines, Palau, Yap, and PNG (West Pacific). This separation into three groups is also evident in the pairwise *F*_ST_ ordination (Fig. 5). The southern Mariana Island samples separate from all other samples along PCoA Axis 1, which explains 62.2% of the variation in the data, whereas the East Micronesia and West Pacific groupings separate out along PCoA axis 2, which explains 15.4% of the variation. This pattern is consistent with all analyses, suggesting the southern Mariana Islands is the most genetically differentiated cluster of three populations (southern Mariana Islands, East Micronesia, and the West Pacific) present in the study region.

Assignment tests showed none of 331 individual recruits sampled from Guam could be excluded from originating from the southern Mariana Islands population, however, only seven individuals (2%) were also excluded from both the East Micronesia and West Pacific populations, and thus, could be assigned as originating from the southern Mariana Islands population (Table 5). The East Micronesia population was excluded as a population of origin for recruits more often than the West Pacific (9% vs. 2%), potentially indicating a greater barrier to dispersal between the Southern Mariana Islands and East Micronesia than the West Pacific.

**Table 5:** Probabilities of exclusion for all recruits excluded from one or more of the three populations detected by structure and geneland. Probabilities < 0.05 are shown in bold typeface

			Probability of population membership		
			
Recruit ID	Site	Rec. event	E. Micronesia	S. Mariana Is.	W. Pacific
SS0235	Achang	November 2007	**0**.**023**	0.254	0.138
SS0243	Achang	November 2007	**0**.**020**	0.093	0.122
SS0224	Agat	November 2007	**0**.**006**	0.342	0.103
SS0231	Agat	November 2007	**0**.**004**	0.300	**0**.**027**
SS0905	Agat	September 2008	**0**.**007**	0.355	0.077
SS0269	East Agana	November 2007	**0**.**013**	0.431	0.170
SS0947	East Agana	September 2008	**0**.**015**	0.517	0.085
SS0949	East Agana	September 2008	**0**.**036**	0.427	0.146
SS0775	Governors	September 2008	**0**.**039**	0.137	0.267
SS0786	Governors	September 2008	**0**.**001**	0.084	**0**.**002**
SS0802	Governors	September 2008	**0**.**036**	0.327	0.203
SS0286	Ipan	November 2007	**0**.**042**	0.198	0.292
SS0729	Pago	September 2008	**0**.**012**	0.138	**0**.**016**
SS0734	Pago	September 2008	**0**.**011**	0.604	0.090
SS0736	Pago	September 2008	**0**.**008**	0.118	**0**.**026**
SS0755	Pago	September 2008	**0**.**042**	0.472	0.239
SS0528	Rios	July 2008	**0**.**046**	0.316	0.113
SS0826	Rios	September 2008	**0**.**008**	0.385	**0**.**038**
SS0829	Rios	September 2008	**0**.**015**	0.596	0.081
SS0840	Rios	September 2008	**0**.**002**	0.192	**0**.**010**
SS0842	Rios	September 2008	**0**.**012**	0.154	0.073
SS0848	Rios	September 2008	**0**.**028**	0.456	0.138
SS0865	Rios	September 2008	**0**.**008**	0.057	**0**.**038**
SS0542	Tanguisson	July 2008	**0**.**013**	0.477	0.108
SS1028	Tanguisson	October 2008	**0**.**049**	0.293	0.248

E. Micronesia, East Micronesia; S. Mariana Is., southern Mariana Islands; W. Pacific, West Pacific.

### Fine-scale spatiotemporal population structure

Results indicate genetic homogeny across Guam and Saipan both spatially and temporally. Comparisons using AMOVA did not reveal any genetic differentiation between adults and recruits (Table 4B). PCoA and nonparametric MANOVA on pairwise *R*_XY_ values for adults and recruits also showed no significant differences between life-history stages (*F*= 0.11, *P*= 0.71), or sample sites (*F*= 0.62, *P*= 0.74). There was also no evidence of sweepstakes reproduction. Permutation tests in fstat comparing mean heterozygosity and *F*_ST_ between adult and recruit samples revealed no differences between adult and recruit groups (*H*_O_: *P*= 0.08; *F*_ST_: *P*= 0.51). When considering only the recruit samples, there was no difference between the eight sampling sites (*F*= 1.31, *P*= 0.35). There was also no evidence of differentiation between cohorts that settled at different recruitment events using either AMOVA (Table 4C) or pairwise *R*_XY_ analyses (*F*= 2.23, *P*= 0.14), indicating temporal stability of gene flow over the 11-month sampling period.

**Figure 8:**
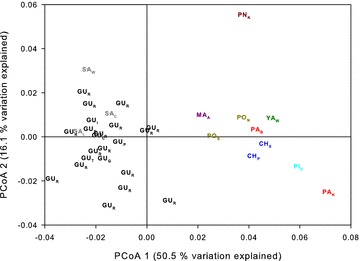
Principal coordinate analysis of all pairwise *F*_ST_ values for all spatial and temporal sample collections for *Siganus spinus*. Both axes combined explain 66.6% of the total variation. Site codes for the region-wide samples are as Table 1, with Guam recruit samples labeled as GuR. Samples are color coded by island. Note that all recruit samples cluster close to the Guam/Saipan adult samples.

Consistent with no evidence of spatial or temporal genetic structure within Guam, when the recruit samples from Guam were added to the region-wide dataset, the PCoA ordination indicated clearly that the Guam recruit samples were most closely related to the Southern Marianas Island cluster (Fig. 8). Moreover, pairwise nonparametric MANOVA comparisons of recruit samples against the three clusters detected by Bayesian analyses showed that the Guam recruit samples were not significantly different from the southern Mariana Island cluster (*t*= 1.84, *P*= 0.133), but were significantly differentiated from the two other region-wide clusters (West Pacific: *t*= 7.66, *P*= 0.001 and East Micronesia: *t*= 5.53, *P*= 0.001). The similarity between Guam recruit samples and the Southern Marianas Island cluster in the region-wide analysis is suggestive of limited gene flow between the Southern Marianas Islands and the rest of the region.

## Discussion

We found significant large-scale spatial genetic structure in *S. spinus* populations across the West Pacific, with the Southern Mariana Islands strongly separated from the rest of the region. The stability of the Southern Mariana Islands population was confirmed by temporal sampling of newly settled fish from four separate recruitment events over a period of 11 months. Recruitment cohorts were genetically indistinguishable from each other and from the resident adult population on Guam and Saipan. These results indicate limited connectivity with the rest of the West Pacific and highlight the likely significance of self-recruitment in maintaining the unique genetic signature of reef fish from the Southern Mariana Islands.

Three genetically distinct populations were found across the region: the southern Mariana Islands (Guam and Saipan), East Micronesia (Chuuk, Pohnpei, and Majuro), and the West Pacific (Philippines, Palau, Yap, and PNG). The Southern Mariana Islands were strongly separated from the rest of the region in all *F*_ST_ and Bayesian analyses, indicating significant isolation from the rest of the region. The East Micronesian and West Pacific populations exhibited less, but nevertheless still significant, differentiation from each other. Genetic structure between populations was detected when using Bayesian analyses with either prior sampling group information (structure) or spatially explicit geographical coordinates (geneland). These models have been criticized for coaxing data into forming cluster groups by weighting results according to sample location. However, in the case of structure, simulation and empirical evidence has shown that the inclusion of sampling information does not lead to detection of genetic structure when genetic structure is not present (Hubisz et al. 2009). Additionally, the trend of isolation-by-distance, which we detected across the region, refutes the hypothesis of a single panmictic population between the Marshall Islands and the Philippines.

Our study is the first to document significant genetic structure between the southern Mariana Islands and its nearest neighbors in Micronesia, and this has potentially profound considerations for future management of reef fish populations in the region. Previous genetics-based studies within the Pacific Ocean basin have generally focused on connectivity from a phylogeographic perspective. These studies used more highly conserved allozyme and mtDNA markers, which reflect historical patterns of connectivity that may not be representative of contemporary patterns of gene flow. Contrasting patterns of genetic structure were nevertheless observed across a range of fish species, although most of the cited studies reported an absence of genetic structure (Bay et al. 2004; Craig et al. 2007; Horne et al. 2008; Gaither et al. 2010). One study, using allozyme markers, did however find strong genetic differentiation across the Pacific, in the surgeonfish *Acanthurus triostegus* (Planes and Fauvelot 2002). Populations from Guam, Philippines, Palau, and the Great Barrier Reef (GBR) were all genetically differentiated from each other but formed a western Pacific clade more similar to each other than the rest of the Pacific. In the only other example of connectivity assessment across Micronesia, Rhodes et al. (2003) found strong genetic structure in the camouflage grouper *Epinephelus polyphekadion* using three microsatellite loci. Cluster analysis identified three regions of genetic differentiation: Pohnpei and the Marshall Islands; New Caledonia and the GBR; and Palau. The separation of Pohnpei and the Marshall Islands from Palau is also evident in *S. spinus* (this study), which could indicate a consistent barrier to dispersal between East and West Micronesia. However, interannual variation was observed within Pohnpei, which may suggest populations of this species are not temporally stable.

In rabbitfish, large variations in patterns of population structure have been observed between species. Using mtDNA, Iwamoto et al. (2009) found no evidence of genetic structure in *S. spinus* among islands in Okinawan waters separated by 430 km. In contrast, *S. guttatus* sampled concurrently did exhibit genetic structuring. The authors attribute this disparity to the early life-history characteristics of *S. spinus* with their larvae having greater dispersive potential than *S. guttatus*. A similar result was obtained when *S. argenteus*, a species with early life-history characteristics similar to *S. spinus* (Woodland 1990; Iwamoto et al. 2009), was examined for genetic structure along the Philippine coast, along with *S. fuscescens* (Magsino and Juinio-Meñez 2008). Genetic structure was present in *S. fuscescens*, but not in *S. argenteus.* Again, this was attributed to early life-history characteristics, and also oceanographic conditions. Further supporting this conclusion, a molecular systematic comparison of eight species of rabbitfish found that *S. argenteus* and *S. spinus* exhibited the lowest intraspecific genetic differentiation of the genus (Lemer et al. 2007). Our results also demonstrate high connectivity over large distances, as demonstrated by the delineation of the East Micronesia population that spans a distance of more than 2000 km. With a pelagic larval duration of 32 days, strong swimming ability and large settlement size, *S. spinus* undoubtedly possess traits that appear to favor long-distance dispersal. However, simple life-history traits have shown to be poor predictors of both genetic differentiation and self-recruitment (Bay et al. 2006; Almany et al. 2007; Weersing and Toonen 2009; but see Riginos and Victor 2001). Also, larval behavior, such as swimming, schooling, and natal homing could actively reduce dispersal (Selkoe et al. 2008). While little is known of *S. spinus* larval behavior, presettlement stage fish have been observed on Guam schooling offshore prior to recruitment onto reefs (Kami and Ikehara 1976).

Oceanographic models have been used to predict levels of dispersal and have shown in many cases that prevailing current regimes are a driving mechanism of connectivity, and, thus, the genetic structure of marine populations (Glig and Hilbish 2003; Galindo et al. 2006; Galarza et al. 2009; White et al. 2010). The southern Mariana Islands are located in the path of the North Equatorial Current (NEC), which flows predominately northwestward at approximately 0.1–0.3 msec^–1^ (Fig. 1). In contrast, all our other sampling locations (except PNG) are located in lower latitudes and are under the influence of the eastward flowing Equatorial Countercurrent. A recent biophysical model constructed using satellite-derived broad-scale current regimes to predict population connectivity of coral larvae, revealed complete isolation of the southern Mariana Islands from the rest of the Pacific when the pelagic larval duration of simulated larvae was set to 30 days (Treml et al. 2008). This pattern was consistent even when accounting for oceanographic variability as a result of El Niño-La Nina events. Their model also predicted high levels of connectivity across the Micronesian islands, which strongly agrees with the large-scale spatial structure we report here; suggesting ocean-scale current flows are a significant influence on patterns of connectivity in *S. spinus*. At the local scale, transient eddies have been observed forming in the lee of Guam, generated by the NEC. These eddies may be sufficiently energetic to return larvae to their natal reefs (Wolanski et al. 2003), revealing a potential mechanism for enhanced self-recruitment within the southern Mariana Islands. A biophysical model recently compiled for Guam has also provided evidence in support of self-entrainment through island eddy formation (A. Halford, pers. comm.).

In contrast to many similar studies, we also investigated the temporal strength of our observed spatial structuring and found no genetic differences between new cohorts and adult *S. spinus* on Guam. We also found no evidence of sweepstakes reproduction as a mechanism for structuring populations: individuals within recruitment events did not (on average) share more alleles with other recruits or with adults from the same site. This suggests the southern Mariana Islands should be managed as a single demographically connected population. Temporal stability amid low levels of gene flow suggests persistent self-recruitment may be responsible for maintaining the distinct genetic structure found across the southern Mariana Islands (Swearer et al. 2002). Further indirect evidence for self-recruitment comes from the PCoA of pairwise *F*_ST_ values for all sample groups. When grouped, allele frequencies from the recruit samples were not differentiated from adult samples from the southern Mariana Islands but showed significant differences when compared to adult populations from both the East Micronesia and West Pacific populations. Direct genetic evidence for self-recruitment in marine reef fish is steadily increasing. Studies using parentage analysis and assignment tests have shown locally produced larvae can be a primary source of population replenishment (Jones et al. 2005; Planes et al. 2009; Christie et al. 2010). Despite Bayesian assignment tests failing to assign most recruits to a single population of origin, the seven recruits that could be assigned all originated from the southern Mariana Islands population, providing some affirmation of self-recruitment as a significant process in structuring the population. The low power of assignment is likely a result of the low number of markers used and their relatively low variability. Empirical and simulation studies have shown low polymorphic loci provide less accurate assignments than high polymorphic loci (Waples and Gaggiotti 2006; Saenz-Agudelo et al. 2009). Reduced polymorphism is a common problem with loci originally designed for a different species (Ellegren et al. 1997; Neff and Gross 2001), and we understand that these limitations may have hindered our ability to assign recruits and detect genetic structure using individual-based analyses at finer resolutions, both spatially and temporally. Unfortunately, financial and logistical constraints prevented the development of species-specific markers in this study and it is anticipated that further insights may be gained from the use of additional loci with greater polymorphism (Kalinowski 20024). However, the life-history characteristics of *S. spinus* may preclude the use of parentage analysis. Rabbitfish are fast growing and subsequently suffer high natural mortality (Ntiba and Jaccarini 1988; Grandcourt et al. 2007). *Siganus spinus* can also reach sexual maturity within approximately 6 months (A. Halford, unpubl. data). Such a dynamic demographic structure may dramatically reduce the chance of finding parent–offspring pairs. Nonetheless, given that, under certain conditions, very few successful migrants per generation can lead to genetic homogeny among populations, our results provide strong evidence for treating the southern Mariana Islands as an isolated, predominately self-recruiting population (Mills and Allendorf 1996).

The results presented in this study may have significant implications for the management of Guam's *S. spinus* fishery. As a species subjected to high fishing pressure and with historically high variance in recruitment, *S. spinus* has been recognized on Guam as a species in need of conservation. Our results suggest a greater emphasis on effective local management of fish stocks is required, because recruitment may be directly linked to the standing stock of local adult *S. spinus*. Moreover, suitable habitat for *S. spinus* is limited in the Northern Mariana Islands as these islands are much younger geologically and do not contain significant reef flat habitat (Houk and Starmer 2010), and are unlikely to be acting as a larval source for this species. Currently, there are five Marine Preserves on Guam, three of which contain reef flat habitat suitable for *S. spinus*. Each of these three preserves contains greater *S. spinus* biomass than adjacent fished sites (A. Halford, unpubl. data). However, the preserves are opened seasonally to allow fishing for newly settled *S. spinus* recruits. The effects of directly fishing recruitment pulses (as in Pauly et al. 1998) within the preserves are unknown, but the results presented here suggest adequate protection of adult spawning stocks may be essential for building resilience in the local population of *S. spinus* and to help ensure the future sustainability of the fishery.

Further research into connectivity within the Mariana archipelago region is clearly needed. If the results presented here are mirrored in other species, then managing for future resilience of local reef fish populations will have to explicitly consider the strength of the stock-recruitment relationship. An integral part of this relationship is the magnitude of larval exchange between islands within the southern Mariana archipelago (Guam and Saipan). While clearly enough to maintain genetic homogeneity, the actual extent of larval exchange between islands remains unknown. Such information is critical for providing effective conservation of exploited marine resources.

## Appendix

**Table A1:** Characteristics of five microsatellite loci from 18 collections of *Siganus spinus* (>60 mm FL) from the western Pacific. Given are: Site code and sample size (*N*), number of alleles (*A*), observed heterozygosity (*H*_O_), expected heterozygosity (*H*_E_), and inbreeding coefficient (*F*_IS_)^1^

Sample code (*N*)		*Sfus-113*	*Sfus-56*	*Sfus-8*	*Sfus-9*	*Sfus-98*
PI_D_	*A*	13	2	19	2	2
88	*H*_O_	0.824	0.011	0.852	0.264	0.138
	*H*_E_	0.851	0.011	0.919	0.229	0.185
	*F*_IS_	0.038	0.000	0.080	–0.147	**0**.**262**
PA_B_	*A*	15	2	18	2	2
50	*H*_O_	0.804	0.040	0.786	0.146	0.163
	*H*_E_	0.858	0.039	0.918	0.170	0.215
	*F*_IS_	0.074	–0.010	**0**.**156**	0.152	0.250
PA_K_	*A*	12	2	15	2	2
40	*H*_O_	0.917	0.050	0.750	0.179	0.175
	*H*_E_	0.845	0.049	0.890	0.242	0.160
	*F*_IS_	–0.070	–0.013	0.171	0.271	–0.083
YA_W_	*A*	9	2	13	2	2
26	*H*_O_	0.760	0.154	0.692	0.192	0.269
	*H*_E_	0.793	0.142	0.894	0.233	0.286
	*F*_IS_	0.062	–0.064	**0**.**244**	0.194	0.079
PN_K_	*A*	8	2	9	2	2
9	*H*_O_	0.875	0.111	0.667	0.222	0.111
	*H*_E_	0.820	0.105	0.852	0.198	0.401
	*F*_IS_	0.000	0.000	0.273	–0.067	0.750
GU_C_	*A*	9	2	12	1	3
26	*H*_O_	0.846	0.038	0.583	0.000	0.269
	*H*_E_	0.798	0.038	0.852	0.000	0.240
	*F*_IS_	–0.041	0.000	**0**.**334**	NA	–0.101
GU_G_	*A*	8	2	12	1	2
39	*H*_O_	0.744	0.026	0.447	0.000	0.205
	*H*_E_	0.730	0.025	0.896	0.000	0.184
	*F*_IS_	–0.005	0.000	**0**.**511**	NA	–0.101
GU_I_	*A*	12	2	15	2	3
67	*H*_O_	0.800	0.015	0.621	0.015	0.258
	*H*_E_	0.772	0.015	0.889	0.015	0.275
	*F*_IS_	–0.028	0.000	**0**.**310**	0.000	0.072
GU_P_	*A*	11	2	12	1	2
28	*H*_O_	0.821	0.107	0.583	0.000	0.250
	*H*_E_	0.783	0.101	0.856	0.000	0.219
	*F*_IS_	–0.032	–0.039	**0**.**337**	NA	–0.125
GU_T_	*A*	12	2	13	2	3
87	*H*_O_	0.720	0.048	0.579	0.011	0.184
	*H*_E_	0.769	0.046	0.895	0.011	0.187
	*F*_IS_	0.070	–0.018	**0**.**359**	0.000	0.020
SA_C_	*A*	8	2	11	1	3
18	*H*_O_	0.750	0.111	0.647	0.000	0.333
	*H*_E_	0.768	0.105	0.808	0.000	0.285
	*F*_IS_	0.055	–0.030	**0**.**228**	NA	–0.140
SA_L_	*A*	10	2	13	1	3
56	*H*_O_	0.745	0.055	0.648	0.000	0.268
	*H*_E_	0.753	0.053	0.891	0.000	0.236
	*F*_IS_	0.019	–0.019	**0**.**281**	NA	–0.126
SA_W_	*A*	9	2	10	2	2
19	*H*_O_	0.684	0.105	0.500	0.053	0.368
	*H*_E_	0.715	0.100	0.859	0.051	0.361
	*F*_IS_	0.070	–0.029	**0**.**444**	0.000	0.008
CH_P_	*A*	12	1	16	2	2
92	*H*_O_	0.800	0.000	0.525	0.258	0.228
	*H*_E_	0.789	0.000	0.910	0.273	0.202
	*F*_IS_	–0.009	NA	**0**.**428**	0.058	–0.124
CH_X_	*A*	12	1	12	2	2
51	*H*_O_	0.780	0.000	0.405	0.362	0.216
	*H*_E_	0.799	0.000	0.876	0.347	0.192
	*F*_IS_	0.034	NA	**0**.**547**	–0.032	–0.111
PO_S_	*A*	12	1	15	2	2
114	*H*_O_	0.738	0.000	0.524	0.167	0.257
	*H*_E_	0.786	0.000	0.902	0.181	0.237
	*F*_IS_	0.066	NA	**0**.**423**	0.086	–0.080
PO_N_	*A*	13	1	14	2	2
108	*H*_O_	0.800	0.000	0.522	0.279	0.241
	*H*_E_	0.799	0.000	0.898	0.254	0.265
	*F*_IS_	0.004	NA	**0**.**424**	–0.095	0.097
MA_A_	*A*	10	1	12	2	2
53	*H*_O_	0.792	0.000	0.413	0.113	0.377
	*H*_E_	0.816	0.000	0.878	0.107	0.306
	*F*_IS_	0.038	NA	**0**.**537**	–0.051	–0.224

1 Numbers in boldface type indicate significant departure from Hardy–Weinberg equilibrium at the 0.05 level after Bonferroni correction.

**Table A2:** Characteristics of six microsatellite loci from samples of *Siganus spinus* recruits and adults (>100 mm FL) from Guam. Given are: Collection code and sample size (*N*), number of alleles (*A*), observed heterozygosity (*H*_O_), expected heterozygosity (*H*_E_), and inbreeding coefficient (*F_IS_)*^1^

Sample code (*N*)		*Sfus-113*	*Sfus-56*	*Sfus-8*	*Sfus-9*	*Sfus-98*	*Sfus-21*
AchN7	*A*	10	1	11	1	3	6
20	*H*_O_	0.737	0.000	0.471	0.000	0.100	0.750
	*H*_E_	0.706	0.000	0.841	0.000	0.096	0.713
	*F*_IS_	0.070	NA	**0**.**554**	NA	–0.013	–0.027
AgaN7	*A*	6	2	12	2	2	5
13	*H*_O_	0.846	0.077	0.538	0.077	0.154	0.692
	*H*_E_	0.722	0.074	0.882	0.074	0.142	0.707
	*F*_IS_	–0.133	0.000	**0**.**423**	0.000	–0.044	0.061
AgaS8	*A*	9	2	13	1	2	7
20	*H*_O_	0.900	0.050	0.700	0.000	0.100	0.700
	*H*_E_	0.806	0.049	0.858	0.000	0.095	0.748
	*F*_IS_	–0.091	0.000	**0**.**208**	NA	–0.027	0.089
CocA	*A*	9	2	12	2	3	7
24	*H*_O_	0.875	0.042	0.565	0.042	0.333	0.542
	*H*_E_	0.803	0.041	0.857	0.041	0.288	0.616
	*F*_IS_	–0.069	0.000	**0**.**394**	0.000	–0.136	0.142
EAgN7	*A*	8	2	8	1	2	6
16	*H*_O_	1.000	0.125	0.429	0.000	0.125	0.813
	*H*_E_	0.801	0.117	0.829	0.000	0.117	0.693
	*F*_IS_	–0.218	–0.035	**0**.**582**	NA	–0.035	–0.140
EAgS8	*A*	10	1	13	2	3	6
21	*H*_O_	0.762	0.000	0.429	0.048	0.238	0.762
	*H*_E_	0.787	0.000	0.887	0.046	0.214	0.662
	*F*_IS_	0.056	NA	**0**.**534**	0.000	–0.087	–0.127
GovA	*A*	8	2	13	1	3	7
26	*H*_O_	0.769	0.038	0.542	0.000	0.423	0.577
	*H*_E_	0.737	0.038	0.891	0.000	0.341	0.616
	*F*_IS_	–0.024	0.000	**0**.**461**	NA	–0.222	0.083
GovO8	*A*	8	2	10	2	2	7
20	*H*_O_	0.737	0.050	0.444	0.050	0.100	0.550
	*H*_E_	0.745	0.049	0.847	0.049	0.095	0.626
	*F_IS_*	**0**.**113**	0.000	**0**.**556**	0.000	–0.027	0.147
GovS8	*A*	9	2	11	1	2	6
26	*H*_O_	0.808	0.038	0.478	0.000	0.115	0.538
	*H*_E_	0.752	0.038	0.882	0.000	0.174	0.531
	*F*_IS_	–0.054	0.000	**0**.**541**	NA	0.353	0.006
IpaA	*A*	11	2	14	2	3	6
42	*H*_O_	0.805	0.024	0.615	0.024	0.167	0.732
	*H*_E_	0.761	0.024	0.889	0.024	0.194	0.682
	*F*_IS_	–0.006	0.000	**0**.**375**	0.000	0.151	–0.013
IpaN7	*A*	8	2	10	2	2	5
13	*H*_O_	0.727	0.077	0.500	0.077	0.077	0.462
	*H*_E_	0.802	0.074	0.833	0.074	0.074	0.444
	*F*_IS_	**0**.**299**	0.000	**0**.**489**	0.000	0.000	0.000
IpaS8	*A*	10	1	10	1	2	6
19	*H*_O_	0.789	0.000	0.368	0.000	0.316	0.579
	*H*_E_	0.755	0.000	0.853	0.000	0.266	0.683
	*F*_IS_	–0.019	NA	**0**.**586**	NA	–0.161	0.178
PagA	*A*	9	2	6	1	2	5
11	*H*_O_	0.818	0.091	0.500	0.000	0.364	0.545
	*H*_E_	0.789	0.087	0.765	0.000	0.298	0.636
	*F*_IS_	0.011	0.000	**0**.**468**	NA	–0.177	0.189
PagS8	*A*	9	1	13	1	3	5
22	*H*_O_	0.864	0.000	0.667	0.000	0.136	0.636
	*H*_E_	0.774	0.000	0.872	0.000	0.129	0.661
	*F*_IS_	–0.093	NA	**0**.**299**	NA	–0.033	0.061
RioJ8	*A*	8	1	10	1	2	7
15	*H*_O_	0.733	0.000	0.333	0.000	0.200	0.600
	*H*_E_	0.773	0.000	0.880	0.000	0.180	0.562
	*F*_IS_	0.086	NA	**0**.**642**	NA	–0.077	–0.033
RioO8	*A*	5	1	8	1	1	5
8	*H*_O_	0.800	0.000	0.375	0.000	0.000	0.500
	*H*_E_	0.740	0.000	0.836	0.000	0.000	0.609
	*F*_IS_	**0**.**398**	NA	**0**.**596**	NA	NA	0.243
RioS8	*A*	12	1	14	1	3	6
48	*H*_O_	0.771	0.000	0.548	0.000	0.292	0.563
	*H*_E_	0.733	0.000	0.894	0.000	0.259	0.641
	*F*_IS_	–0.041	NA	**0**.**478**	NA	0.463	0.134
TanA	*A*	10	2	13	1	3	7
33	*H*_O_	0.719	0.030	0.594	0.000	0.152	0.697
	*H*_E_	0.793	0.030	0.900	0.000	0.142	0.685
	*F_IS_*	0.149	0.000	**0**.**377**	NA	–0.053	–0.003
TanJ8	*A*	8	1	8	1	3	7
19	*H*_O_	0.632	0.000	0.474	0.000	0.263	0.789
	*H*_E_	0.673	0.000	0.845	0.000	0.234	0.716
	*F*_IS_	0.089	NA	**0**.**461**	NA	–0.098	–0.076
TanN7	*A*	8	1	7	1	2	4
9	*H*_O_	0.778	0.000	0.556	0.000	0.222	0.667
	*H*_E_	0.784	0.000	0.796	0.000	0.198	0.574
	*F*_IS_	0.067	NA	0.355	NA	–0.067	–0.103
TanO8	*A*	8	1	11	1	2	6
23	*H*_O_	0.826	0.000	0.545	0.000	0.000	0.652
	*H*_E_	0.787	0.000	0.870	0.000	0.083	0.680
	*F*_IS_	–0.027	NA	**0**.**425**	NA	**1**.**000**	0.063
TangS8	*A*	8	1	11	2	2	7
19	*H*_O_	0.789	0.000	0.526	0.053	0.105	0.737
	*H*_E_	0.770	0.000	0.875	0.051	0.188	0.652
	*F*_IS_	0.002	NA	**0**.**421**	0.000	–0.116	–0.103

1 Numbers in bold typeface indicate significant departure from Hardy–Weinberg equilibrium at the 0.05 level after Bonferroni correction.

## References

[b1] Almany GR, Berumen ML, Thorrold SR, Planes S, Jones GP (2007). Local replenishment of coral reef fish populations in a marine reserve. Science.

[b2] Anderson M (2001). A new method for non-parametric multivariate analysis of variance. Austral Ecol.

[b3] Atema J, Kingsford MJ, Gerlach G (2002). Larval reef fish could use odour for detection, retention and orientation to reefs. Mar. Ecol. Prog. Ser.

[b4] Balloux F, Lugon-Moulin N (2002). The estimation of population differentiation with microsatellite markers. Mol. Ecol.

[b5] Bassler PC, Aguon CF (2006).

[b6] Bay LK, Choat JH, van Herwerden L, Robertson DR (2004). High genetic diversities and complex genetic structure in an Indo-Pacific tropical reef fish (*Chlorurus sordidus*): evidence of an unstable evolutionary past?. Mar. Biol.

[b12] Bay LK, Crozier RH, Caley MJ (2006). The relationship between population genetic structure and pelagic larval duration in coral reef fishes on the Great Barrier Reef. Mar. Ecol. Prog. Ser.

[b13] Caley M, Carr M, Hixon M (1996). Recruitment and the local dynamics of open marine populations. Annu. Rev. Ecol. Syst.

[b14] Chapuis M, Estoup A (2007). Microsatellite null alleles and estimation of population differentiation. Mol. Biol. Evol.

[b15] Chirichetti PR (1996).

[b16] Christie M (2010). Parentage in natural populations: novel methods to detect parent-offspring pairs in large data sets. Mol. Ecol. Res.

[b17] Christie MR, Johnson DW, Stallings CD, Hixon MA (2010). Self-recruitment and sweepstakes reproduction amid extensive gene flow in a coral-reef fish. Mol. Ecol.

[b18] Clarke K, Gorley R (2006).

[b19] Cowen RK, Sponaugle S (2009). Larval dispersal and marine population connectivity. Annu. Rev. Mar. Sci.

[b20] Cowen RK, Lwiza KMM, Sponaugle S, Paris CB, Olson DB (2000). Connectivity of marine populations: open or closed?. Science.

[b21] Craig MT, Eble JA, Bowen BW, Robertson DR (2007). High genetic connectivity across the Indian and Pacific Oceans in the reef fish *Myripristis berndti* (Holocentridae). Mar. Ecol. Prog. Ser.

[b22] Earl DA, von Holdt BM (2011). structure harvester: a website and program for visualizing structure output and implementing the Evanno method. Conserv. Genet. Res.

[b23] Ellegren H, Moore S, Robinson N (1997). Microsatellite evolution—a reciprocal study of repeat lengths at homologous loci in cattle and sheep. Mol. Biol. Evol.

[b24] Evanno G, Regnaut S, Goudet J (2005). Detecting the number of clusters of individuals using the software STRUCTURE: a simulation study. Mol. Ecol.

[b25] Excoffier L, Laval G, Schneider S (2005). Arlequin (version 3.0): an integrated software package for population genetics data analysis. Evol. Bioinform. Online.

[b26] Excoffier L, Smouse P, Quattro J (1992). Analysis of molecular variance inferred from metric distances among DNA haplotypes: application to human mitochondrial DNA restriction data. Genetics.

[b27] Falush D, Stephens M, Pritchard J (2007). Inference of population structure using multilocus genotype data: dominant markers and null alleles. Mol. Ecol. Notes.

[b28] Fisher R (2005). Swimming speeds of larval coral reef fishes: impacts on self-recruitment and dispersal. Mar. Ecol. Prog. Ser.

[b29] Gaines S, Gaylord B, Gerber L, Hastings A, Kinlan B (2007). Connecting places: the ecological consequences of dispersal in the sea. Oceanography.

[b30] Gaither M, Toonen R, Robertson D, Planes S, Bowen B (2010). Genetic evaluation of marine biogeographical barriers: perspectives from two widespread Indo-Pacific snappers (*Lutjanus kasmira* and *Lutjanus fulvus*. J. Biogeogr.

[b31] Galarza JA, Carreras-Carbonell J, Macpherson E (2009). The influence of oceanographic fronts and early-life-history traits on connectivity among littoral fish species. Proc. Natl. Acad. Sci. U.S.A.

[b32] Galindo HM, Olson DB, Palumbi SR (2006). Seascape genetics: a coupled oceanographic-genetic model predicts population structure of Caribbean corals. Curr. Biol.

[b33] Gerlach G, Atema J, Kingsford MJ, Black KP, Miller-Sims V (2007). Smelling home can prevent dispersal of reef fish larvae. Proc. Natl. Acad. Sci. U.S.A.

[b34] Gilg MR, Hilbish TJ (2003). The geography of marine larval dispersal: coupling genetics with fine-scale physical oceanography. Ecology.

[b35] Goudet J (2001). http://www2.unil.ch/popgen/softwares/fstat.htm.

[b36] Grandcourt E, Al Abdessalaam T, Francis F, Al Shamsi A (2007). Population biology and assessment of the white-spotted spinefoot, *Siganus canaliculatus* (Park, 1797), in the southern Arabian Gulf. J. Appl. Ichthyol.

[b37] Guillot G, Estoup A, Mortier F, Cosson J (2005). A spatial statistical model for landscape genetics. Genetics.

[b38] Guillot G, Santos F, Estoup A (2008). Analysing georeferenced population genetics data with Geneland: a new algorithm to deal with null alleles and a friendly graphical user interface. Bioinformatics.

[b39] Hedgecock D, Barber PH, Edmands S (2007). Genetic approaches to measuring connectivity. Oceanography.

[b40] Hedgecock D (2010). Determining parentage and relatedness from genetic markers sheds light on patterns of marine larval dispersal. Mol. Ecol.

[b41] Hellberg ME (2007). Footprints on water: the genetic wake of dispersal among reefs. Coral Reefs.

[b42] Hepburn RI, Sale PF, Dixon B, Heath DD (2009). Genetic structure of juvenile cohorts of bicolor damselfish (*Stegastes partitus*) along the Mesoamerican barrier reef: chaos through time. Coral Reefs.

[b43] Hixon M, Pacala S, Sandin S (2002). Population regulation: historical context and contemporary challenges of open vs. closed systems. Ecology.

[b44] Horne JB, van Herwerden L, Choat JH, Robertson DR (2008). High population connectivity across the Indo-Pacific: congruent lack of phylogeographic structure in three reef fish congeners. Mol. Phylogenet. Evol.

[b45] Houk P, Starmer J (2010). Constraints on the diversity and distribution of coral-reef assemblages in the volcanic Northern Mariana Islands. Coral Reefs.

[b46] Hubisz M, Falush D, Stephens M, Pritchard J (2009). Inferring weak population structure with the assistance of sample group information. Mol. Ecol. Res.

[b47] Iwamoto K, Takemura A, Yoshino T, Imai H (2009). Molecular ecological study of *Siganus spinus* and *S. guttatus* from Okinawan waters based on mitochondrial DNA control region sequences. J. Oceanogr.

[b48] Jones DB, Jerry DR, McCormick MI, Bay LK (2010). The population genetic structure of a common tropical damselfish on the Great Barrier Reef and eastern Papua New Guinea. Coral Reefs.

[b49] Jones GP, Almany GR, Russ GR (2009). Larval retention and connectivity among populations of corals and reef fishes: history, advances and challenges. Coral Reefs.

[b50] Jones GP, Milicich MJ, Emslie MJ, Lunow C (1999). Self-recruitment in a coral reef fish population. Nature.

[b51] Jones GP, Planes S, Thorrold SR (2005). Coral reef fish larvae settle close to home. Curr. Biol.

[b52] Kalinowski SK (2002). How many alleles per locus should be used to estimate genetic distances?. Heredity.

[b53] Kami H, Ikehara I (1976). Notes on the annual juvenile siganid harvest in Guam. Micronesia.

[b54] Kritzer J, Sale P (2004). Metapopulation ecology in the sea: from Levins’ model to marine ecology and fisheries science. Fish Fish.

[b55] Lecchini D, Planes S, Galzin R (2005). Experimental assessment of sensory modalities of coral-reef fish larvae in the recognition of their settlement habitat. Behav. Ecol. Sociobiol.

[b56] Leis JM, Carson-Ewart BM, Cato DH (2002). Sound detection in situ by the larvae of a coral-reef damselfish (Pomacentridae). Mar. Ecol.

[b57] Leis JM, Sale PF (1991). The pelagic phase of coal reef fishes: larval biology of coral reef fishes. The ecology of fishes on coral reefs.

[b58] Lemer S, Aurelle D, Vigliola L, Durrand J-D, Borsa P (2007). Cytochrome b barcoding, molecular systematics and geographic differentiation in rabbitfishes (Siganidae). C. R. Biol.

[b59] Li G, Hedgecock D (1998). Genetic heterogeneity, detected by PCR-SSCP, among samples of larval Pacific oysters *(Crassostrea gigas)* supports the hypothesis of large variance in reproductive success. Can. J. Fish. Aquat. Sci.

[b60] Magsino RM, Juinio-Meñez MA (2008). The influence of contrasting life history traits and oceanic processes on genetic structuring of rabbitfish populations *Siganus argenteus* and *Siganus fuscescens* along the eastern Philippine coasts. Mar. Biol.

[b61] Man A, Law R, Polunin NVC (1995). Role of marine reserves in recruitment to reef fisheries: A metapopulation model. Biol. Conserv.

[b62] Manel S, Gaggiotti O, Waples R (2005). Assignment methods: matching biological questions with appropriate techniques. Trends Ecol. Evol.

[b63] McArdle B, Anderson M (2001). Fitting multivariate models to community data: a comment on distance-based redundancy analysis. Ecology.

[b64] Mills LS, Allendorf FW (1996). The one-migrant-per-gerneration rule in conservation and management. Conserv. Biol.

[b65] Mora C, Sale PF (2002). Are populations of coral reef fish open or closed?. Trends Ecol. Evol.

[b66] Neff B, Gross M (2001). Microsatellite evolution in vertebrates: inference from AC dinucleotide repeats. Evolution.

[b67] Neigel JE (1997). A comparison of alternative strategies for estimating gene flow from genetic markers. Annual Rev. Ecol. Syst.

[b68] Newton K, Côté IM, Pilling GM, Jennings S, Dulvy NK (2007). Current and Future Sustainability of Island Coral Reef Fisheries. Curr. Biol.

[b69] Ntiba MJ, Jaccarini V (1988). Age and growth parameters of *Siganus sutor* in Kenyan marine inshore water, derived from numbers of otolith microbands and fish lengths. J. Fish Biol.

[b70] Pauly D, Christensen V, Dalsgaard J, Froese R, Torres F (1998). Fishing Down Marine Food Webs. Science.

[b71] Peakall R, Smouse PE (2006). GENALEX 6: genetic analysis in Excel. Population genetic software for teaching and research. Mol. Ecol. Notes.

[b72] Pearse DE, Crandall KA (2004). Beyond F-ST: analysis of population genetic data for conservation. Conserv. Genet.

[b73] Peatkau D, Slade R, Burden M, Estoup A (2004). Genetic assignment methods for the direct, real-time estimation of migration rate: a simulation-based exploration of accuracy and power. Mol. Ecol.

[b74] Piry S, Alapetite A, Cornuet L-M, Paetkau D, Baudouin L, Estoup A (2004). GENECLASS2: A Software for Genetic Assignment and First-Generation Migrant Detection. J. Hered.

[b75] Planes S, Fauvelot C (2002). Isolation by distance and vicariance drive genetic structure of a coral reef fish in the Pacific Ocean. Evolution.

[b76] Planes S, Lenfant P (2002). Temporal change in the genetic structure between and within cohorts of a marine fish, *Diplodus sargus*, induced by a large variance in individual reproductive success. Mol. Ecol.

[b77] Planes S, Jones GP, Thorrold SR (2009). Larval dispersal connects fish populations in a network of marine protected areas. Proc. Natl. Acad. Sci. U.S.A.

[b78] Pritchard J, Stephens M, Donnelly P (2000). Inference of population structure using multilocus genotype data. Genetics.

[b79] Purcell JFH, Cowen RK, Hughes CR, Williams DA (2006). Weak genetic structure indicates strong dispersal limits: a tale of two coral reef fish. Proc. R. Soc. B.

[b80] Queller D, Goodnight K (1989). Estimating relatedness using genetic markers. Evolution.

[b81] Rannala B, Mountain JL (1997). Detecting immigration by using multilocus genotypes. Proc. Natl. Acad. Sci. U.S.A.

[b82] Ravago-Gotanco R, Lumibao C, Pante M (2010). Isolation and characterization of thirteen microsatellite markers for the rabbitfish, *Siganus fuscescens*. Conserv. Genet. Res.

[b83] Raymond M, Rousset F (1995). genepop (version 3.4): population genetics software for exact tests and ecumenicism. J. Hered.

[b84] Rhodes KL, Lewis RI, Chapman RW, Sadovy Y (2003). Genetic structure of camouflage grouper, *Epinephelus polyphekadion* (Pisces: Serranidae), in the western central Pacific. Mar. Biol.

[b85] Riginos C, Victor BC (2001). Larval spatial distributions and other early life-history characteristics predict genetic differentiation in eastern Pacific blennioid fishes. Proc. R. Soc. Lond. Ser. B.

[b86] Roughgarden J, Iwasa Y, Baxter C (1985). Demographic theory for an open marine population with space-limited recruitment. Ecology.

[b87] Saenz-Agudelo P, Jones GP, Thorrold SR, Planes S (2009). Estimating connectivity in marine populations: an empirical evaluation of assignment tests and parentage analysis under different gene flow scenarios. Mol. Ecol.

[b88] Selkoe KA, Henzler CM, Gaines SD (2008). Seascape genetics and the spatial ecology of marine populations. Fish Fish.

[b89] Selkoe KA, Gaines SD, Caselle JE, Warner RR (2006). Current shifts and kin aggregation explain genetic patchiness in fish recruits. Ecology.

[b90] Simpson SD, Meekan M, Montgomery J, McCauley R, Jeffs A (2005). Homeward sound. Science.

[b91] Slatkin M (1993). Isolation by distance in equilibrium and non-equilibrium populations. Evolution.

[b92] Stobutzki IC, Bellwood DR (1997). Sustained swimming abilities of the late pelagic stages of coral reef fishes. Mar. Ecol.

[b93] Swearer SE, Shima JS, Hellberg ME (2002). Evidence of self-recruitment in demersal marine populations. Bull. Mar. Sci.

[b94] Treml EA, Halpin PN, Urban DL, Pratson LF (2008). Modeling population connectivity by ocean currents, a graph-theoretic approach for marine conservation. Landscape Ecol.

[b95] Tsuda RT, Bryan PG (1973). Food preference of juvenile *Siganus rostratus* and *S. spinus* in Guam. Copeia.

[b96] Underwood JN, Smith LD, Van Oppen MJH, Gilmour JP (2007). Multiple scales of genetic connectivity in a brooding coral on isolated reefs following catastrophic bleaching. Mol. Ecol.

[b97] van Herwerden L, Benzie J, Davies C (2003). Microsatellite variation and population genetic structure of the red throat emperor on the Great Barrier Reef. J. Fish Biol.

[b98] van Oosterhout C, Hutchinson WF, Wills DPM, Shipley P (2004). MICRO-CHECKER: software for identifying and correcting genotyping errors in microsatellite data. Mol. Ecol. Notes.

[b99] Victor BC, Wellington GM (2000). Endemism and the pelagic larval duration of reef fishes in the eastern Pacific Ocean. Mar. Ecol.

[b100] Waples RS, Gaggiotti O (2006). What is a population? An empirical evaluation of some genetic methods for identifying the number of gene pools and their degree of connectivity. Mol. Ecol.

[b101] Warner RR, Cowen RK (2002). Local retention of production in marine populations: evidence, mechanisms, and consequences. Bull. Mar. Sci.

[b102] Weersing K, Toonen RJ (2009). Population genetics, larval dispersal, and connectivity in marine systems. Mar. Ecol.

[b103] White C, Selkoe K, Watson J (2010). Ocean currents help explain population genetic structure. Proc. R. Soc. B.

[b104] Whitlock M, McCauley D (1999). Indirect measures of gene flow and migration: *F*_ST_≠ 1/(4*Nm*+ 1). Heredity.

[b105] Wolanski E, Richmond RH, Davis G, Deleersnijder E, Leben RR (2003). Eddies around Guam, an island in the Mariana Islands group. Cont. Shelf Res.

[b106] Woodland DJ (1990). Revision of the fish family Siganidae with descriptions of two new species and comments on distribution and biology. Indo-Pacific Fishes.

[b107] Zeller D, Booth S, Davis G, Pauly D (2007). Re-estimation of small-scale fishery catches for US flag-associated island areas in the western Pacific: the last 50 years. Fish. Bull.

